# Obliterative cholangiopathy in acquired cystic biliary atresia type III after cyst perforation: a case report

**DOI:** 10.1186/s12887-018-1125-8

**Published:** 2018-05-11

**Authors:** Tsugumichi Koshinaga, Kensuke Ohashi, Kakou Ono, Hide Kaneda, Takeshi Furuya

**Affiliations:** Department of Pediatric Surgery, Nihon University, School of Medicine, 30-1 Ooyaguchi-kamicho, Itabashi-ku, Tokyo, 173-8610 Japan

**Keywords:** Biliary atresia, Acquired biliary atresia, Biliary perforation, Biliary cyst, Obliterative cholangiopathy

## Abstract

**Background:**

In biliary atresia, the disease process of obliterative cholangiopathy may begin in the perinatal period; however, no chronological evidence exists on how the cholangiopathy progresses to biliary obliteration.

This is the first acquired case with the final diagnosis of type III cystic biliary atresia with an extrahepatic biliary cyst which showed the progression of obliterative cholangiopathy in chronological order after birth.

**Case presentation:**

An 81-day-old girl presented with acute abdominal distress due to bilious peritonitis caused by biliary cyst perforation, for which she underwent emergency biliary drainage. Postoperative images showed a dilated common bile duct and hepatic ducts bilaterally, with flow of the contrast medium to the duodenum through the dilated common bile duct. Biochemistry of the bile collected during and after the operation revealed elevated levels of pancreatic enzymes in the bile from the gallbladder. The patient was diagnosed as having a congenital choledochal cyst and underwent laparotomy at 120 days of age which revealed that she had pancreaticobiliary maljunction. The biliary cyst was resected at the narrow portion just above the junction with the main pancreatic duct. During dissection up to the hepatic hilum, we found that the hilar hepatic ducts were bilaterally replaced by fibrous tissue and were obstructed, leading to a diagnosis of type III a1, μ biliary atresia. The fibrous tissue was excised, and hepatic portoenterostomy was performed according to the Kasai procedure. The patient’s postoperative course was uneventful and the jaundice resolved within 1 month. She has had normal liver function tests with no episode of cholangitis for 3 years after discharge.

**Conclusions:**

We demonstrated the process of acquired type III biliary atresia in a patient with cystic biliary atresia and biliary cyst perforation. To the best of our knowledge, this is the first case of acquired cystic biliary atresia showing chronological progression of the course of obliterative cholangiopathy, providing a better understanding of the development of type III biliary atresia as an acquired disease.

## Background

Most patients with biliary atresia still have greenish stools shortly after birth. The disease process of obliterative cholangiopathy may begin in the perinatal period [1]; however, no evidence exists on the chronological progression of cholangiopathy to biliary obliteration.

We describe here a case with the final diagnosis of acquired type III biliary atresia with a perforated extrahepatic biliary cyst which showed the chronological progression of obliterative cholangiopathy after birth. This is the first clinical case showing the disease process of acquired biliary atresia described in a previous report [2].

## Case presentation

An 81-day-old girl was transferred to the accident and emergency center of Nihon University Itabashi Hospital from her local pediatrician’s clinic because of marked abdominal distention and convulsions with loss of consciousness. The convulsions subsided with intravenous administration of diazepam and midazolam; however, the abdominal distension worsened, and was referred to pediatric surgery for surgical indication due to the intraperitoneal ascitic fluid collection. She was previously healthy, with appropriate growth and development and no history of trauma to her abdomen, after birth by normal vaginal delivery to a 38-year-old secundigravida mother at 37 weeks of gestation. The mother was noted to have a past history of extrahepatic bile duct resection with hepaticojejunostomy due to pancreaticobiliary maljunction along with a congenital choledochal cyst.

On physical examination, the patient was found to be drowsy. Her temperature was 36.7 °C, and oxygen saturation was 98% while breathing oxygen at a flow rate of 10 L/min. Her weight was 4590 g and height was 55 cm. Her abdomen was markedly distended, with tense, shiny skin. Guarding and tenderness over the entire abdomen were also noted on palpation. Her sclerae appeared anicteric.

**Fig. 1:**
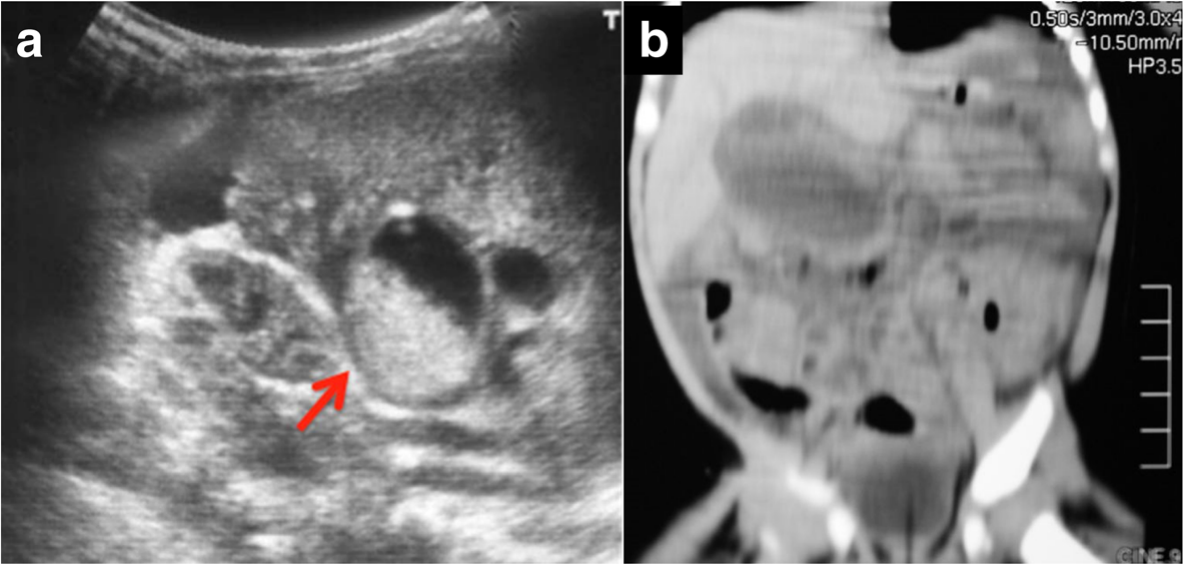
Abdominal ultrasound and CT images. Ultrasonography (Panel **a**) showed a cystic lesion in the hepatic hilum, with accumulated fluid and debris. CT scan (Panel **b**) showed extrahepatic bile duct dilatation with massive ascites

Blood and serum biochemical evaluations revealed leukocytosis (19,400/μL) and elevated total bilirubin (2.41 mg/dl), direct bilirubin (1.02 mg/dl), aspartate aminotransferase (72 IU/l), alkaline phosphatase (1371 IU/l), lactate dehydrogenase (LDH) (362 IU/l), and creatine kinase (CK) (371 IU/l) levels. The levels of alanine aminotransferase (17 IU/l) and C-reactive protein (CRP) (0.10 mg/dl) were within normal limits. Venous gas analysis indicated a pH of 7.357, bicarbonates (HCO_3_^−^) of 16.3 mmol//l, and base excess (BE) of − 9.5 mmol/l. Abdominal ultrasonography revealed the presence of massive ascites and a cystic lesion at the hepatic hilum measuring 3 cm in diameter, with collected fluid and debris. The cystic lesion appeared to be connected to the intrahepatic bile duct, suggesting a dilated extrahepatic duct (Fig. 1a). Abdominal CT scan revealed extrahepatic bile duct dilatation with massive ascites (Fig. 1b).

### Clinical course after admission

**Fig. 2:**
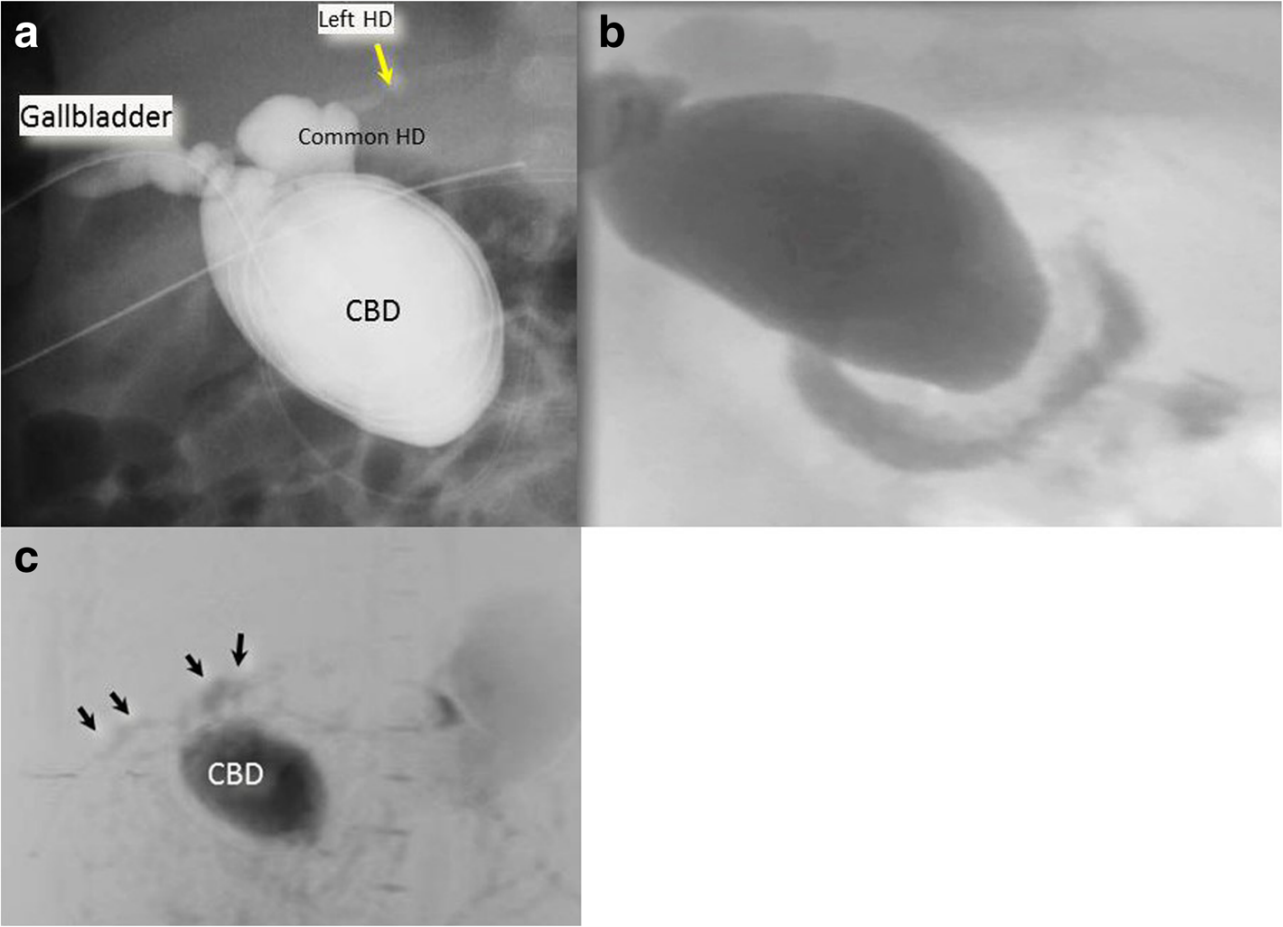
Postoperative cholangiography. Cholangiography performed via a catheter placed in the common bile duct (Panel **a**) showed only the left hepatic duct and no flow of the contrast medium into the duodenum through the dilated common bile duct. However, digitized video-fluoroscopy cholangiography (Panel **b**) found evidence of biliary patency, showing flow of the contrast agent into the duodenum. MRCP (Panel **c**) depicted the dilated common bile duct and bilateral hepatic ducts, although the junction of the pancreatic duct and choledochus was obscure

The patient underwent emergency laparotomy with a diagnosis of biliary peritonitis due to perforation of a congenital choledochal cyst. Laparotomy findings revealed massive biliary ascites in the peritoneum, but no abnormalities of the liver and gallbladder on gross appearance. The common bile duct appeared as a cyst 3 cm in diameter in the hepatoduodenal ligament. We could not find the exact site of biliary perforation despite careful exploration. Then, we performed cholangiography of the entire extrahepatic biliary tract by inserting a small catheter tapping from the gallbladder to the common bile duct via the cystic duct**,** which also failed to identify the site of biliary perforation. We avoided excessive pressure when injecting the contrast medium during cholangiography. No passage of contrast medium into the duodenum was shown. The final diagnosis of minimal biliary perforation was made at that time. The operative procedure was accomplished by inserting an indwelling drainage catheter into the dilated common bile duct via the cystic duct and by intraperitoneal irrigation. The patient successfully recovered from the critical situation with good drainage of bile from the dilated common bile duct. The daily output of bile was between 50 to 100 ml. After she became in a stable condition, cholangiography via the catheter placed in the common bile duct was performed. It initially showed only the left hepatic duct, and no passage of the contrast medium into the duodenum through the dilated common bile duct (Fig. 2a). However, digitized video-fluoroscopy cholangiography revealed evidence of biliary patency, demonstrating the flow of contrast into the duodenum (Fig. 2b). Magnetic resonance choledocopancreatography (MRCP) scan depicted the dilated common bile duct and bilateral hepatic ducts, although the junction of the pancreatic duct and choledochus was obscure (Fig. 2c). These images failed to identify the pancreaticobiliary maljunction. However, biochemistry of the bile collected during and after the operation revealed that the levels of pancreatic enzymes, such as phospholipase A2, were elevated in bile from the gallbladder and the common bile duct (Table 1). This evidence was highly suggestive of pancreaticobiliary maljunction. The patient was diagnosed as having a congenital choledochal cyst; hence, surgery including extrahepatic bile duct resection was scheduled at the age of 120 days.

**Table 1:** Chemistry of bile collected during and after operation

	Intraoperative collection			Postoperative collection^a^
	Ascites	Gallbladder	Common bile duct	Gallbladder via drainage catheter
Total bilirubin (mg/dl)	9.5	n.a.	n.a.	n.a.
Amylase (IU/l)	n.a.	29	4	380
Trypsin (ng/ml)	n.a.	2200	n.a.	n.a.
PhospholipaseA2 (ng/dl)	2040	6370	157,000	n.a.
Elastase 1 (ng/dl)	560	1300	63,000	2,400,000
Lipase (U/l)	114	267	9958	n.a.

n.a, not available

^a^Bile collected on the second day after operation

**Fig. 3:**
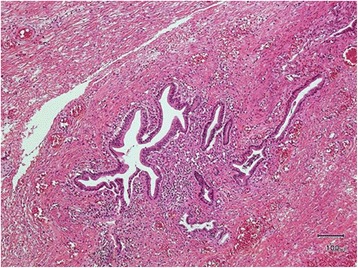
Histopathologic findings of liver (Hematoxylin eosin × 200). Liver biopsy shows a few inflammatory cells infiltrated in the periportal area presenting slight fibrosis, but no significant ductular proliferation noted

During the second laparotomy, dissection along the dilated choledochal cyst to the intrapancreatic bile duct revealed a narrow portion between the distal part of the bile duct and pancreatic duct—the pancreaticobiliary maljunction— in the pancreatic parenchyma. Intraoperative cholangiography showed no communication between the common bile duct and the pancreatic duct at the operation. The distal end of the dilated biliary cyst was ligated and transected at the narrow portion just above its junction with the main pancreatic duct. Dissecting along to the hepatic hilum, we found that the hilar hepatic ducts had been bilaterally replaced by fibrous tissue and were obstructed, leading to a diagnosis of type III a1, μ biliary atresia. The fibrous tissue was excised, and hepatic portoenterostomy was performed according to the Kasai procedure. Histopathologic examination showed that most of the lining epithelium of the resected biliary cyst was desquamated. Liver biopsy shows a few inflammatory cells infiltrated in the periportal area presenting slight fibrosis, but no significant ductular proliferation noted (Fig. 3). The patient’s postoperative course was uneventful. She has had normal liver function tests with no episodes of cholangitis for 3 years since her discharge from the hospital.

## Discussion and conclusions

We demonstrated here the pathophysiological process of acquired type III biliary atresia in a patient with cystic biliary atresia with biliary cyst perforation. To the best of our knowledge, this is the first case of acquired biliary atresia showing the chronological progression in the course of obliterative cholangiopathy.

**Fig. 4:**
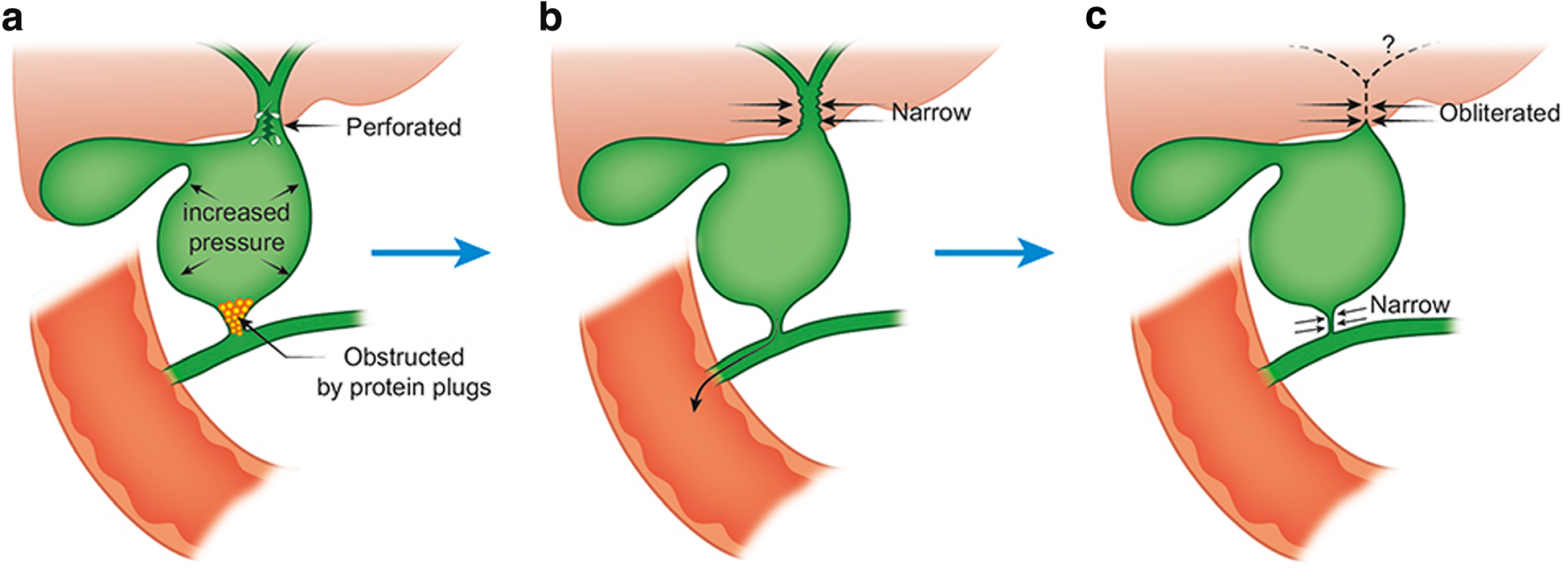
Schema of the disease process together with possible etiologies. Biliary perforation secondary to increased luminal pressure in the biliary cyst due to possible obstruction by protein plugs in the distal end of the common bile duct at 81 days old (Panel **a**). The resultant decrease in intraluminal pressure, together with adequate drainage of the common bile duct and spontaneous removal of the protein plugs contributes to healing the site of perforation (Panel **b**). On the hepatic side, the perihilar bile ducts and common hepatic duct become progressively narrower, marking the beginning of development of biliary atresia. Finally, the perihilar bile ducts become completely obliterated and the distal side of the common bile duct becomes narrower, as was observed during the surgery at 120 days old (Panel **c**)

The case presents evidence that type III biliary atresia develops as an acquired disease. Although acquired biliary atresia was first described in 1996 [2], little is known about the process of obliteration of the biliary tree. The cause of acquired biliary atresia remains unknown. Multifactorial mechanisms may be involved in the etiology of development of obliterative cholangiopathy. The proposed etiologies of acquired biliary atresia possibly include infection [3] and inflammatory responses [4], but rarely include genetic aberrations [5] and developmental malformations [6]. The onset of obliteration of the biliary tree also remains unknown, as in most other types of biliary atresia. The most accepted view is that infants with biliary atresia once had intact biliary trees during fetal life, following which the biliary tree began to obliterate as a secondary phenomenon [7], a rational concept that is yet to be proved by clinical observation of the disease. A recent report has described the pathology of biliary atresia could begin in utero as well as perinatally given the elevation of direct bilirubin soon after birth [8]. If the obliterative cholangiopathy consistent with biliary atresia seen in this case at 120 days of age began in the newborn period it certainly progressed very slowly. The reason for the biliary perforation not occurring until 81 days of life is unclear. The present case with type III acquired biliary atresia demonstrates the process of postnatal obliteration of the biliary tree, as shown in the chronological schema of the disease process (Fig. 4). The pathological process possibly occurs in the following sequence. First, biliary perforation occurs due to increased intraluminal pressure in the biliary cyst due to possible obstruction by protein plugs at the distal end of the common bile duct. Second, the resultant decrease in intraluminal pressure in the cyst secondary to adequate drainage of the common bile duct via the indwelling catheter and by spontaneous removal of the protein plugs, with subsequent restoration of the patency of the distal end of the common bile duct, facilitates healing of the site of perforation. On the hepatic side, the perihilar bile ducts and the common hepatic duct become progressively narrower at the beginning of the development of the biliary atresia. Finally, the perihilar bile ducts become completely obliterated, and the distal end of the common bile duct becomes narrow**,** as was seen during the second laparotomy in our patient.

Our case represents cystic biliary atresia, consisting of an extrahepatic biliary cyst with biliary atresia, in which development of the cyst was the primary event rather than secondary to increased luminal pressure due to biliary obstruction. The biliary cyst formed before the hilar biliary obliteration on the distal side seen in the present case. From a clinical standpoint, Davenport [9] divided biliary atresia into four broad groups: syndromic biliary atresia, cystic biliary atresia, cytomegalovirus-associated biliary atresia, and isolated biliary atresia. Among these subgroups, cases with cystic biliary atresia, an uncommon and clinically distinct variant of biliary atresia, have been previously reported by many authors. One of the previous reports [10] mentioned that 21 (72%) of the 29 cases with cystic biliary atresia in their clinical series were found to be type III biliary atresia. According to the national registry of the Japanese Biliary Atresia Society, 131 (32%) of 408 cases with cystic biliary atresia were classified as type III atresia (1989-2015; http://jbas.net/registration/). Among those 131 cases, 31 cases (24%) had yellow or slightly yellow bile, providing evidence of the communication between the cyst lumen and intrahepatic biliary tree. The registry provides no further detailed clinical information, such as the process of development of cystic biliary atresia. One previous report described the following as characteristics of the cystic biliary atresia: diagnosed in the prenatal period, presenting with jaundice from birth, and presence of closed cyst formation in the biliary tree, as confirmed by cholangiography [11]. However, previous reports failed to show the etiology of type III cystic biliary atresia, and lacked chronological evidence of development of the disease [11–13]. It is accepted that type III cystic biliary atresia has a different pathogenesis as compared to atresia without biliary cyst formation. The evidence that the cyst develops distal to obstruction of the hilar biliary tree cannot be explained only by an increase of intraluminal pressure, as in our case with type III cystic biliary atresia. In the present case, biliary cyst formation clearly preceded the hilar biliary obliteration, as seen in chronological cholangiograms. The etiological difference between biliary cysts associated with acquired biliary atresia and congenital choledochal cyst perforation, therefore, remains uncertain.

Our case presents perforation of a biliary cyst as the initial manifestation of acquired biliary atresia. The role of biliary perforation in the process of obliteration of the biliary tree in the present case with type III biliary atresia remains unknown. Biliary perforation is a rare condition of various etiologies, such as pancreaticobiliary maljunction with and/or without a choledochal cyst, developmental weakness of the biliary wall, trauma, viral infection, stenosis of the biliary tract, and distal obstruction of the extrahepatic bile duct [14, 15]. Among these proposed etiologies, pancreaticobiliary maljunction has been focused on as the most probable etiological hypothesis [16, 17]. In the present case, pancreaticobiliary maljunction was found during the second operation. Biliary perforation with this congenital anomaly is associated with an abrupt increase in intramural pressure of the biliary tree secondary to impaction of protein plugs [16]. We suspect that protein plugs or inspissated bile were the possible cause of obstruction in the present case, which may have resulted in distal biliary obstruction that resolved following adequate biliary drainage [18]. Our patient’s mother had a history of surgery for pancreaticobiliary maljunction. However, it still remains unknown whether to etiologically associate this history with the onset of biliary atresia in our patient. The combination of a mother with pancreaticobiliary maljunction and daughter with biliary atresia has not been previously reported. Further, the site of biliary perforation in the present patient with type III biliary atresia remains unknown, although the site of perforation may have been closely associated with the obliterative process of hilar biliary obstruction. The most common site of biliary perforation is reportedly the anterior wall of the common bile duct, adjacent to its junction with the cystic duct, due to possible embryogenic mural weakness of the common bile duct at its junction with the cystic duct [19, 20]. This mural weakness is vulnerable to ischemia and distal obstruction, resulting in perforation. Some investigators have suggested that extravasated bile in adjacent periductal tissues could lead to protracted inflammation and fibrosis, causing secondary obliteration of the hilar biliary tract [7].

Biliary atresia may also be caused by failure of the remodeling process at the hepatic hilum, leading to persistence of fetal bile ducts poorly supported by mesenchyme. As bile flow increases perinatally, bile leakage from these abnormal ducts may trigger an intense inflammatory reaction, with subsequent obliteration of the biliary tree [21]. Biliary perforation may occur at the site of embryogenic mural weakness in the abnormal hilar biliary tract, and the leaked bile may play a role in obliteration of the biliary tract. A review of the previous literature found only one case of cystic biliary atresia with biliary perforation reported by Davenport et al. (Table 2). This previous case differed from our case in terms of gestational age at delivery, birth weight, associated conditions, primary symptoms, and age at presentation of primary symptoms. Their case was the first case of type III biliary atresia ever reported. The outcome was fair, without jaundice and with normal liver function tests postoperatively.

**Table 2:** Reported cases with acquired biliary atresia presenting with biliary perforation

Case no.	References	Year reported	Sex	Gestation week at delivery	Birth weight (g)	Associated conditions	Primary symptoms	Age at presentation	Age at suregry	Type of biliary atresia	Site of perforation	Operation perfprmed	Outcome	Years after operation
1	Davenport et al. [2]	1991	Female	28 weeks	800	RDS, PDA	Jaundice, acholic stools, failure to thrive	24 weeks	8 months	I cyst	Pinhole perforation of CBD	local excision of the teretic segment followed by biliary anastomosis	Jaundice free with normal liver function tests	4 years
2	Present case	2013	Female	37 weeks	2560	Epilepsy	Abdominal distention	81 days	81 days	III	Not confirmed	Portoenterostomy	Jaundice free with normal liver function tests	3 years

*RDS* respiratory distress syndrome, *PDA* Patent ductus arteriosus, *CBD* common bile duct

Ours is the first case demonstrating the pathophysiological process of acquired type III biliary atresia in a patient with a perforated biliary cyst, providing a better understanding of the development of type III biliary atresia as an acquired disease.

## Abbreviations

BE: Base excess
CK: Creatine kinase
CRP: C-reactive protein
CT: Computed tomography
HCO_3_: Bicarbonates
LDH: Lactate dehydrogenase
MRCP: Magnetic resonance cholangiopancreatography
